# Development of a miniature device for emerging deep brain stimulation paradigms

**DOI:** 10.1371/journal.pone.0212554

**Published:** 2019-02-21

**Authors:** Scott D. Adams, Kevin E. Bennet, Susannah J. Tye, Michael Berk, Abbas Z. Kouzani

**Affiliations:** 1 Deakin University, School of Engineering, Geelong, Victoria, Australia; 2 Division of Engineering, Mayo Clinic, Rochester, MN, United States of America; 3 Queensland Brain Institute, the University of Queensland, St Lucia QLD, Australia; 4 Deakin University, School of Medicine, IMPACT SRC, Barwon Health, Geelong, Victoria, Australia; University of California Los Angeles, UNITED STATES

## Abstract

Deep brain stimulation (DBS) is a neuromodulatory approach for treatment of several neurological and psychiatric disorders. A new focus on optimising the waveforms used for stimulation is emerging regarding the mechanism of DBS treatment. Many existing DBS devices offer only a limited set of predefined waveforms, mainly rectangular, and hence are inapt for exploring the emerging paradigm. Advances in clinical DBS are moving towards incorporating new stimulation parameters, yet we remain limited in our capacity to test these in animal models, arguably a critical first step. Accordingly, there is a need for the development of new miniature, low-power devices to enable investigation into the new DBS paradigms in preclinical settings. The ideal device would allow for flexibility in the stimulation waveforms, while remaining suitable for chronic, tetherless, biphasic deep brain stimulation. In this work, we elucidate several key parameters in a DBS system, identify gaps in existing solutions, and propose a new device to support preclinical DBS. The device allows for a high degree of flexibility in the output waveform with easily altered shape, frequency, pulse-width and amplitude. The device is suitable for both traditional and modern stimulation schemes, including those using non-rectangular waveforms, as well as delayed feedback schemes. The device incorporates active charge balancing to ensure safe operation, and allows for simple production of custom biphasic waveforms. This custom waveform output is unique in the field of preclinical DBS devices, and could be advantageous in performing future DBS studies investigating new treatment paradigms. This tetherless device can be easily and comfortably carried by an animal in a back-mountable configuration. The results of in-vitro tests are presented and discussed.

## Introduction

Deep brain stimulation (DBS) is a neurosurgical intervention with well-established therapeutic benefits in several neurological and psychiatric disorders [1, 2]. The treatment is still evolving in two key areas: expansion of the number of disorders treated, and improvements in the technology, including stimulation parameter optimisation. This requires the development of new and improved tools specifically designed to enable studies into new DBS treatment paradigms. Much of this research is occurring at the pre-clinical stage utilizing animal models to test hypotheses prior to human use. This critical step, in turn, is dependent on development of translationally relevant small animal devices.

Progress in this area includes moving towards applying non-rectangular stimulation waveforms or actively altering stimulation parameters during treatment, enabling adaptation of the technology to the changing states of the brain [3, 4]. Most existing clinical and pre-clinical DBS devices cannot perform this type of investigation as producing non-rectangular, or actively altering stimulation parameters is beyond their current design capability. Most current DBS devices produce only a fixed current, and those with adjustable current amplitude often cannot be adjusted through software and require a user to physically interact with potentiometers on the device to update the stimulation parameters [5]. These devices are unsuitable for DBS schemes which utilize non-rectangular waveforms and require changes in the pulse amplitude mid-pulse. This necessitates the need for development of a novel DBS device designed specifically to produce these new DBS waveforms in a form-factor suitable for preclinical animal trials. Our new device can produce a large variety of biphasic stimulation waveforms, and still apply traditional treatment paradigms. Highly customised non-rectangular stimulation schemes can be uploaded to this device via the existing communication port allowing for investigation and refinement of new stimulation types, targets, and methods of treatment.

### Design considerations and constraints

A new DBS device should aim for exceeding the existing devices in as many of the therapeutically relevant parameters as possible while retaining design advances of the most recent iterations. This allows for investigation into how changing these parameters can alter therapeutic outcomes. Further, several additional design considerations are required due to the specific conditions in which pre-clinical testing takes place. These parameters and design considerations are discussed in the following.

#### Stimulation method

Historically, the only method of stimulation available was voltage-controlled stimulation, which applied a voltage pulse of a fixed amplitude to the target neural tissue. However, there is one significant disadvantage with this technique, the lack of control over the charge delivered to the neural tissue. Fakhar, Hastings [6] describes DBS charge density using:

$$ChargeDensity=\frac{Voltage\times Pulsewidth}{Area\times Impedance}=\frac{Current\times Pulsewidth}{Area}$$ (1)

As can be observed in Eq [1], if the stimulation voltage is fixed, the charge density becomes dependant on the neurological impedance. This can be a disadvantage as studies have reported that the impedance of neural tissue can significantly alter over time, and particularly in the period immediately after implantation [7, 8]. This is the key reason that constant voltage stimulation has been mostly superseded by constant current stimulation. By delivering a fixed current stimulation, the charge density becomes independent of neurological impedance changes. Additionally, from the equation it is evident that two other key factors define the intensity of the applied neural stimulation: amplitude of voltage/current applied, and pulse-width.

#### Amplitude

The amplitude of the stimulation pulse is the change in magnitude of the pulse from the zero point. It defines the area of stimulation within the neural tissue. As the amplitude increases, neural elements at increasing distances are exposed to the stimulation [9]. Constant voltage mode stimulation is used from 1–10.5 V in amplitude with the most common use at around 3.6 V, while constant current mode stimulation is used at an amplitude of between 50–500 μA with 200 μA being the most common [10–13].

#### Pulse-width

The pulse-width of the stimulation pulse is the interval for which a single pulse is active. Traditionally DBS treatments have been undertaken with a pulse-width of 60 μs. However, some studies have demonstrated therapeutic potential using pulse-widths of as short as 20 μs, and as long as 450 μs [14–16].

#### Frequency

**Fig 1:**
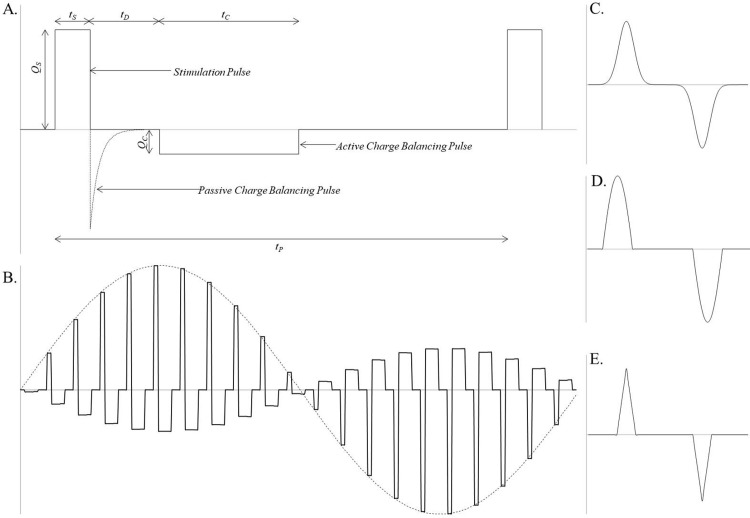
A. Standard stimulation pulse train with active charge balancing. The amplitude of the stimulation pulse Qs can either be a voltage or current pulse depending on the stimulation method. The variable ts is the duration of the pulse, common values are 60 μs or 90 μs. td is the inter-pulse interval, the amount of time between the stimulation pulse and the charge balanced pulse. Qc is the amplitude of the active charge balanced pulse and tc is the period, this pulse can either be symmetric or asymmetric. Finally, tp is the period between pulses, this parameter is the inverse of the pulse frequency. B. An example of a non-constant pulse train, in this case a delayed feedback scheme. C. Gaussian stimulation waveform. D. Sine-wave stimulation waveform. E. Triangular stimulation waveform.

The frequency of the stimulation pulse is defined as the number of pulses per second, see Fig 1. Although not affecting the charge density, the frequency of the stimulation pulse is a significant parameter when examining the total amount of energy delivered to the brain during stimulation Koss Adam, Alterman Ron [17]:

$$TEED=\frac{Voltage^{2}\times Pulsewidth\times Frequency}{Impedance}\times 1Second$$ (2)

As can be observed from Eq (2), the total electrical energy delivered is equally dependant on the stimulation frequency as it is on the pulse-width. Two common frequency schemes are used in DBS studies: Low Frequency Stimulation (LFS) and High Frequency Stimulation (HFS). The HFS is the most common scheme and is undertaken at 130–185 Hz, while the LFS is a newer method which has demonstrated therapeutic potential and is delivered at frequencies below 60 Hz and even as low as 5 Hz [13, 18–20].

#### Waveform & charge balancing

DBS systems include a method of balancing the charge injected into the neural tissue, through injecting an inverse charge into the tissue after the main pulse. Unbalanced pulses can cause significant and permanent damage to the tissue. Testing performed by Piallat, Chabardès [21] found that using monophasic pulses (without charge balancing) caused lesions within the brain in as little 5 minutes, whereas no damage was not found in charge balanced stimulation. In the ideal case, charge balancing ensures that no residual charge remains in the tissue, eliminating damage caused by accumulated charge.

The simplest form of charge balancing is passive charge balancing, which utilizes a passive in-line capacitor to provide an inverse stimulation pulse to the electrode, depicted in Fig 1A. This is an effective method of balancing the injected charge, and is electronically simple to implement. The key disadvantage of this scheme is the lack of control over the output waveform. Several studies have found that different therapeutic effects can be observed by altering the inter-pulse interval, the amplitude and duration of the balancing pulse, or even reversing the order of the pulses [22–25]. This level of control is not achievable using a passive charge balancing scheme.

The active charge balancing scheme employs a controllable circuit to apply reverse polarity pulses to the tissue. These pulses can be symmetric or asymmetric and can be activated in a specific duration (called the inter-pulse interval) after the initial pulse.

Most devices found in the literature with active charge balancing utilized a switching method to invert the polarity of the pulses [5, 26]. Typically, their output is driven through a H-Bridge, which allows the polarity to be reversed by activating / deactivating a transistor pair, inverting the direction of current flow. The disadvantage of this technique is that it requires the inclusion of four switching transistors or a H-Bridge chip, which increases the number of components required, as well as the programming complexity. The result is that in multiple channel systems, each channel requires a H-bridge [26]. Additionally, this method also introduces switching noise when the signal is inverted. A key advantage of the H-Bridge circuit is that the positive and negative pulses will be perfectly matched, simplifying charge balancing. Any system which does not use switching to invert the pulse must either include a feedback mechanism to balance the injected charge or include a passive charge balancing method to ensure that any minor charge mismatch is not causing excessive charge build-up. A method of active charge balancing which can scan between positive and negative stimulation phases without switching, which also includes a passive charge balancing method to correct for pulse mismatch is presented in this work.

The use of pulse shapes other than rectangular pulses in standard DBS has been investigated through a number of simulation studies [27–29]. These studies found that non-rectangular waveforms can potentially achieve the same level of neural activation, with a lower amplitude pulse while being more energy efficient. Increasing the power efficiency of this type of stimulation system can lead to longer battery lifetime, an increased time between charging, and decreased weight / volume of the implanted stimulator. Sinusoidal, triangular and even gaussian waveforms have been analysed for their efficacy. Initial results have been promising, showing some improvements over existing stimulation schemes. However, as the majority of DBS devices are unable to produce these non-rectangular waveform types, there is a lack of experimental studies in this area.

Traditionally the train of pulses delivered to the neural tissue has been consistent, without changes to the amplitude, pulse-width or inter-pulse delay. As such, many devices have been designed with this paradigm in mind, and these parameters have been difficult or impossible to adjust after the stimulation has begun. However, new research is now investigating non-constant stimulation schemes, in particular the efficacy of delayed feedback schemes in disorders with abnormal neuronal synchronization components. These schemes can alter the stimulation amplitude, inter-pulse delay or waveform shape during the stimulation in an arbitrary way (often a sine-wave) which can be altered based on external feedback, see Fig 1B [30–32].

#### Unrestricted movement

As much of the evaluation of preclinical animal trials is through behavioural observation, any device designed for use in this context should be as unrestrictive as possible. If the animal can perform all its daily activities without constraints, the changes caused by the treatment should be simpler to identify and quantify. This can be achieved through: making the device small and lightweight enough to be head/back-mountable or implantable.

#### Cost & reusability

As DBS is often run over a period of days or weeks with many repeated trials, research devices should be as low cost as possible to increase the number of animals which can be tested simultaneously. Additionally, a device should ideally also be reusable and reprogrammable to enable many different chronic experiments to be performed using the same device on different animals.

#### Usability

The device should have an easy to use interface so that researchers can easily: program, activate, and deactivate the stimulation as per their own research requirements. This is an important consideration as researchers in this field are often not trained in altering electronic circuits or updating embedded programs and requiring these skills to change the device operation would be prohibitive to research.

## Programmable device

We present a programmable brain stimulation device, specifically designed for preclinical applications with easily usable active charge balancing. The device can output fully biphasic DBS pulses on a single channel with amplitudes between ±200 μA at a resolution of 200 nA. It can provide pulses of a length as short as 20 μs and at a frequency of up to 10 kHz.

### Hardware

#### Circuit diagram

**Fig 2:**
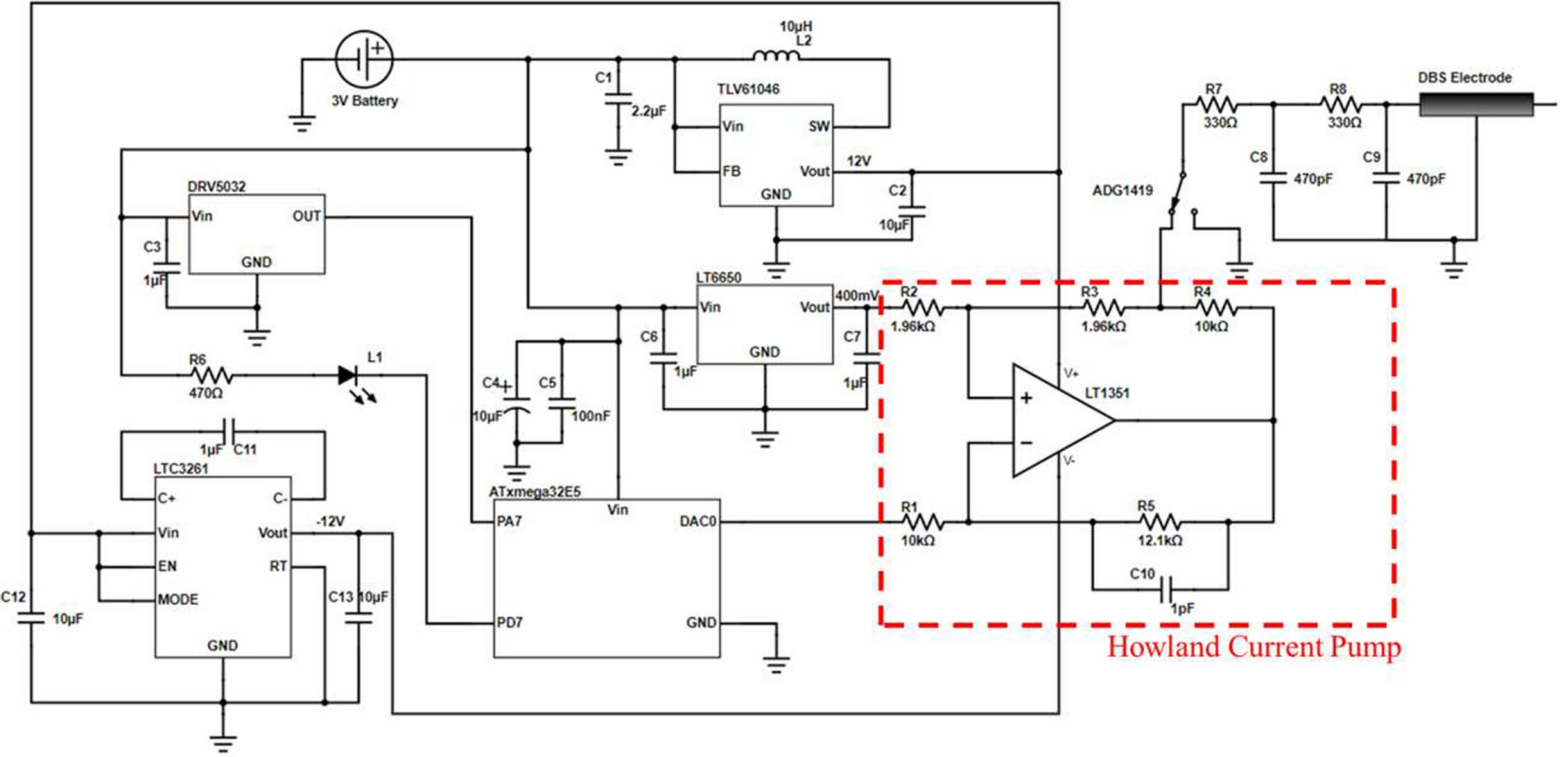
Schematic circuit diagram of the brain stimulation device.

The entire circuit diagram of the device is shown in Fig 2. The diagram shows the interconnection between each of the components of the device. The key components of the circuit are: microcontroller, power source, negative voltage supply, programmable current output, and user interface.

#### Microcontroller

To control the stimulation outputs, implement system logic, allow for programmability, and provide a simple user interface, the ATxmega32E5 8-bit AVR microcontroller was included as the control component in the device. The ATxmega32E5 includes all the standard microcontroller modules such as 32 kB of flash memory, a multi-level interrupt controller, 26 I/O lines and two USART connections, a 12-bit multi-channel 300 kSPS A/D converter. However, it also integrates a highly accurate two-channel 12-bit 1MSPS DAC on-chip [33]. This is a key advantage over other potential options, as utilizing the on-chip functions mean that no external DAC is required, reducing total component count and overall device cost. This device is uniquely suited for miniaturized low-power applications as it is available in a 4 x 4 mm Ultra-Thin Quad Flat No Lead (UQFN) package and integrates several power saving features. The microcontroller can run on voltages as low as 1.6 V and includes an integrated low-power 2 MHz oscillator. Additionally, it includes four sleep modes which can disable peripheral functions to save a considerable amount of power.

#### Power source

The power source selected for this device is a 20 x 3.2 mm CR2032 3V 235 mAh Lithium coin-cell battery. These batteries are low-cost (generally <$2 p/u), robust, suitable in long-term storage, globally available, and have a very stable discharge profile. A non-rechargeable battery was chosen for this device because it allows for a larger capacity, doesn’t require complex charging circuitry and reduces the risk of battery damage due to its tough metal casing. As the device requires approximately 6.5 mA to operate, this battery gives the device ~9 hours of operation. The microcontroller constantly monitors the battery voltage using its the internal features and disables the stimulation once the battery voltage reaches a non-optimum level (<1.8 V). This temporarily activates the on-board LED to alert the user to the state of the stimulator.

#### Voltage supply

In order for the device to apply a consistent and reliable level of stimulation when used on tissue with a wide variety of impedances, an output voltage significantly higher than that supplied by the battery is required. A compliance voltage of 10 V was selected for this device in order to allow for a wide variety of load impedances (up to 50 kΩ at 200 μA).

To create the positive voltage supply rail a boost circuit was implemented which increases the battery voltage to a stable 12 V. This boost circuit is based around the TLV61046A Voltage Boost Converter (Texas Instruments), which can supply a stable 12 V output with an input as small as 1.8 V.

In order for the circuit to provide the inverse pulses required for active charge balancing, a stable negative voltage rail must also be established. This is achieved using a charge-pump voltage inverter. Through charging and discharging a “flying capacitor”, the charge-pump inverter allows for an inverse voltage to be realized from a stable positive supply. Typically, a charge pump inverter is less efficient than its inductive counterpart. However, the fact that it doesn’t require an inductor to operate removes the restrictive layout requirements, reducing the size of the device and increasing its suitability for miniaturization [34]. The charge-pump inverter selected for the device is an LTC3261 developed by Linear Technology (USA). This chip was selected due to its ability to invert the high compliance voltage required as well as for its small power usage (~60 μA). This produces a stable voltage rail of -10 V to power the programmable current output.

#### Programmable current output

Achieving stable, accurate and repeatable current stimulation pulses while retaining full programmability of the stimulation pulse is the key challenge in any DBS system. The presented device utilizes the improved implementation of the Howland Current Pump circuit [35].

This circuit allows for an accurate current source to be created using a single op-amp and five resistors. The typical operation of the Howland Current Pump requires the two feedback paths to both inputs be equal (i.e. the ratios of R1 / R2 and (R3 + R4) / R5 to be equivalent). This circuit allows for the output current to be independent of the load and fully determined by the input voltage levels, and the gains set by the feedback circuits. Assuming the ratios are equivalent, the output current of the basic circuit is determined by the equation *I*_*out*_ *= (Vin*^*+*^*—Vin*^*-*^*) / R3 × R5 / R1*. Utilizing the resistors as shown in Fig 2 (R1 = 10 kΩ, R5 = 12.1 kΩ, R3 = 1.96 kΩ) and a 400 mV precision voltage reference (LT6650) as Vin+, the output current can be adjusted between -375 μA and 250 μA from a DAC output of 0–1 V. This is highly advantageous in the output of a DBS device as it allows for the positive or negative current output to be set, independent of the load from the tissue.

The op-amp selected for this purpose in the device is the LT1351. This op-amp is low-power, has a high slew rate (200 V/μs), a small footprint and can operate accurately on a ±15 V supply. In the device presented in this work, the positive input of the LT1351 is connected to a precision micro-power 400 mV reference chip (LT6650) and the negative input is connected to the DAC0 output of the ATxmega32E5. This allows for a fully controllable output signal between -375 μA and 250 μA. Additionally, an analogue switch chip is included on the PCB to ensure that no transient currents are delivered when the stimulation is inactive.

#### Interface

In order for researchers to interact with the board, a physical interface had to be integrated to enable activation and deactivation of the device with as minimal disturbance to the animal as possible. As such, an ultra-low power hall effect sensor (DRV5032) was included in the system. This means that researchers only need to move a magnet within the active area of the sensor and the microcontroller can enable and disable the stimulation. The user is informed of the change in stimulation status by the activation and deactivation of a blue LED which flashes to alert the user that the status has changed.

#### Stimulation electrode

In order for the stimulation pulse to be injected into the tissue, a specific stimulation electrode must first be implanted. There are several different electrodes available with different functions but the most common one is a stainless-steel twisted wire electrode. The device presented in this study can be easily modified to accept several different electrode types. Bench testing was performed using a single channel twisted stainless-steel electrode.

#### Physical design & construction

**Fig 3:**
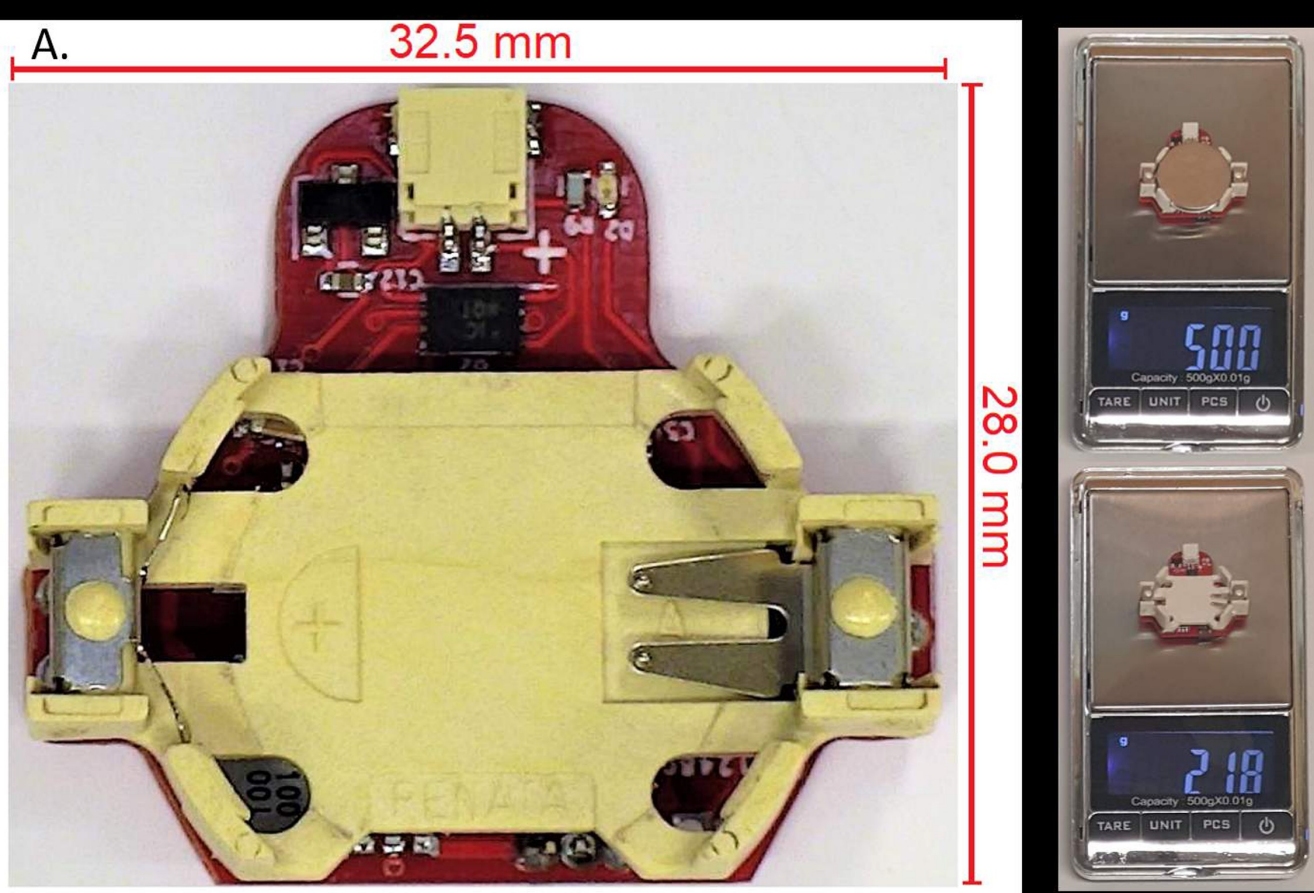
A. A picture of the developed DBS device whose dimensions are 32.5 × 28 × 8 mm. B. The device being weighed with the battery installed. C. The device being weighed with the battery removed.

To implement the circuit of the DBS device, a printed circuit board was designed and fabricated. This circuit board was designed specifically for use in a back-mountable configuration within a rat jacket. The board was designed specifically for use with the selected battery (CR2032) and as such integrates a battery receptacle into the design. The circuit board is fabricated on 0.4 mm thick FR4 in a two-layer configuration and measures approximately 30 mm x 25 mm. The final material cost of the device with all components included is less than $10 USD if produced in large quantities. After fabrication of the boards, the components of the circuit are soldered onto the boards. The circuit board is presented here unsealed, however, sealing the entire device in a waterproof, sterilised material such as silicon is possible if required. Images of the device can be seen in Fig 3.

### Software

The microcontroller is programmed using the C programming language through the Atmel Programming and Debugging Interface using the Atmel ICE programmer. This interface allows for rapid progress on the programming as well as live debugging of the program during development. The CPU frequency is first set to 2 MHz to reduce power usage while still maintaining enough speed to perform the DBS functions. The unused peripherals are then disabled, these include I^2^C interfaces, SPI interfaces, the on-chip EDMA controller, and the event system. The LTC3261 includes an enable/disable pin, and to turn on the negative voltage reference for the circuit this pin must be set low, accomplished through clearing the microcontroller pin PD0. The DAC is then enabled, this involves: setting the output pin data direction (PA2), setting the DAC reference to the internal 1V reference and enabling the output. In this step the output is also set to the ground reference value to avoid any charge entering the electrode prior to the stimulation pulse. The timer interrupt is then enabled to control the waveform. Finally, the on-board LED is flashed several times to indicate that the device is activated, and the microcontroller enters the idle sleep mode to conserve power during stimulation.

To output a customised waveform, the DAC converts a 12-bit value into a voltage level between 0–1 V, and feeds this into the Howland Current Pump circuitry, implemented with the LT1351. The exact waveform output is specified within the code and executed by the timer interrupt. The period of this timer interrupt is altered within the code to reflect the required amount of time before the next change of output is required. For instance: for a 130 Hz waveform with a 200 μA, 90 μs stimulation pulse, a 100 μA, 180 μs charge balanced pulse, and a 1 ms inter-pulse delay, the timer interrupt period is set to 90 μs (for the stimulation pulse), 1ms (for the inter-pulse delay), 180μs (for the charge balanced pulse) and 6.44 ms (for the time before the next pulse), with the DAC output updating at each stage to create the required stimulation amplitude.

To load the program to the device, a serial interface has been included. This interface allows the user to connect a computer to the device through a USB-serial port to upload the program that the stimulator will use. Using this interface, the user can select the frequency, pulse width, pulse shape and amplitude of stimulation from a list of parameters pre-programmed on the device (square, sine, gaussian and triangular). If custom waveforms are required, they can be programmed into the device using the Atmel ICE programmer, through the Program and Debug Interface (PDI) pins on the rear of the device. Once the program has been set, the user can begin the stimulation using the on-board hall effect sensor.

## Evaluation

After the fabrication of the DBS device was complete, the function of the device in several test scenarios was evaluated. These tests consisted of three key stages of in-vitro testing: waveform output, output regulation, verification of electrode shorting, and a stimulation test in physiological saline.

### Waveform output test

**Fig 4:**
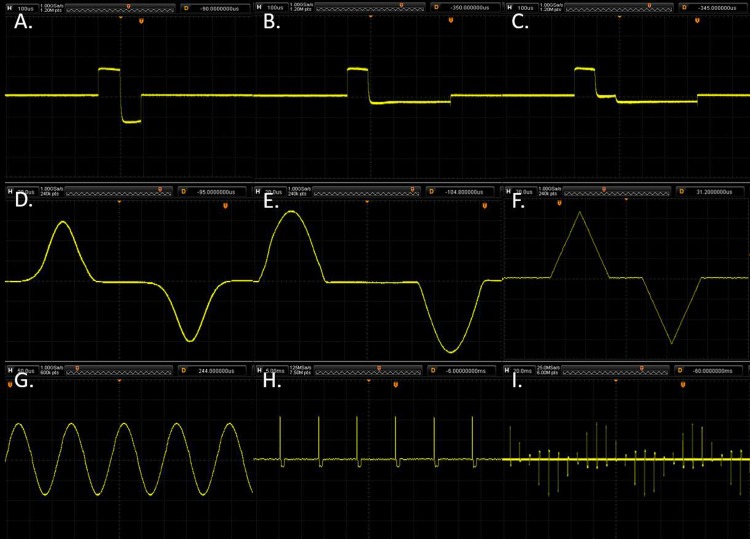
Sample outputs of the DBS device across a 3 kΩ resistive load demonstrating the flexibility of the output. A. A typical symmetric DBS pulse with active charge balancing, 130 Hz, 90 μs, 200 μA stimulation and 90 μs, -200 μA charge balancing. B. An asymmetric charge balanced pulse with an inter-pulse delay, 130 Hz, 90 μs, 200 μA stimulation and 360 μs, -50 μA charge balancing with a 90 μs inter-pulse delay. D. A Gaussian stimulation pulse with 200 μA amplitude at 130 Hz with a 50 μs inter-pulse delay. E. A sinewave stimulation pulse with 200 μA amplitude at 130 Hz with a 50 μs inter-pulse delay. F. A triangular stimulation pulse with 200 μA amplitude at 130 Hz with a 50 μs inter-pulse delay. G. A continuous sinewave with amplitude 200 μA and frequency 10 kHz. H. A typical stimulation pulse-train using the stimulation pulse from Fig 4C. I. A pulsatile delayed feedback stimulation example using the pulse shape in Fig 4C.

The very first test completed on the device was an investigation into the ability of the device to output a variety of DBS waveforms over a simple resistive load. The device was connected across a 2 kΩ resistive load and was programmed to output several different waveforms to demonstrate the flexibility of the device. All measurements were taken by using a RIGOL DS1054Z 50MHz Digital Oscilloscope and can be observed in Fig 4.

### Output regulation test

**Fig 5:**
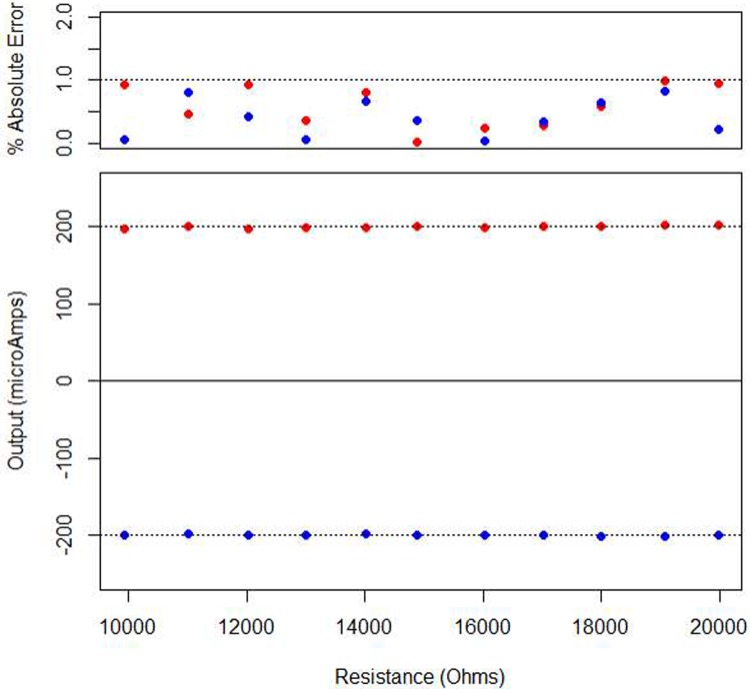
The results of the output regulation experiment. The results clearly show that the circuit successfully outputted both current amplitudes (200 and -200 μA) with regulation of 99%.

The device was tested to determine the effectiveness of the output circuitry at regulating the current being delivered to the load over a variety of resistive load scenarios. The purpose of this test is to evaluate the effectiveness of the current regulation over a variety of test loads. To accomplish this, the device was connected to a variety of resistive loads ranging from 10 to 20 kΩ. These values were selected as they imitate the range of resistance values found by Ewing, Porr [36] and Badstuebner, Stubbe [37] when testing the impedance of small animal DBS electrodes. For any DBS experiment to be successful, the device must output the expected stimulation despite changes in the electrode or brain impedance. This test was performed at both the 200 μA and -200 μA stimulation amplitudes to show regulation in both the positive and negative output spectrums. The results of the experiment are presented in Fig 5.

### Verification of electrode shorting test

**Fig 6:**
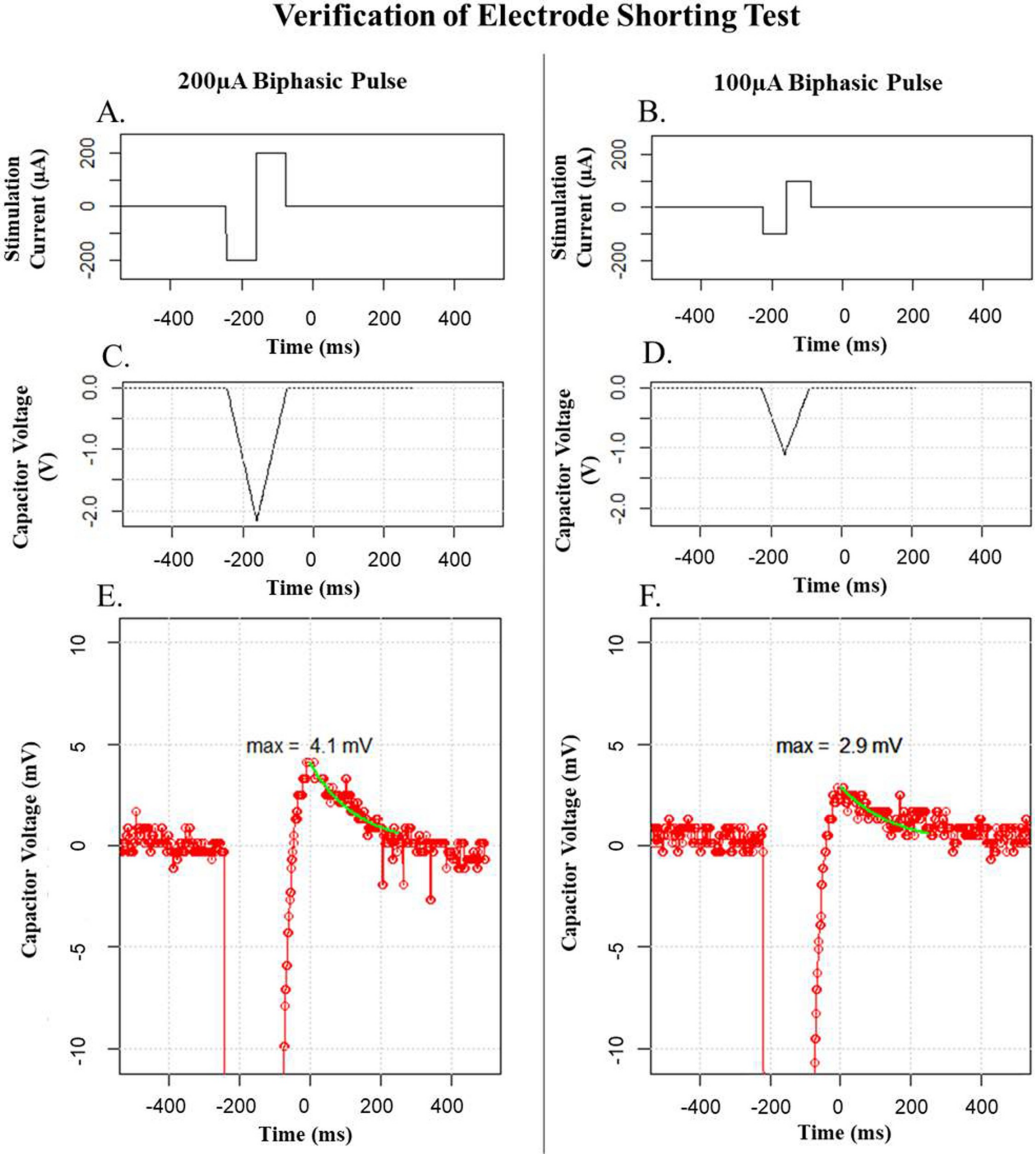
Oscilloscope captures of the return current across a 10 nF capacitor at the shorting phase of a biphasic stimulation pulse. The measured τ of the 200 μA pulse was 131.7 μs and the 100 μA pulse was 155.7 μs. A. The input waveform for the 200 μA pulse test. B. The input waveform for the 100 μA pulse test. C. The voltage across the capacitor in the 200 μA pulse test. D. The voltage across the capacitor in the 100 μA pulse test. E. The voltage across the capacitor in the 200 μA pulse test shown on a smaller scale so the charge balancing can be observed. F. The voltage across the capacitor in the 100 μA pulse test shown on a smaller scale so the charge balancing can be observed.

The third test completed was a verification of the shorting phase of the stimulation. This stage is key in ensuring device safety as it allows any minor charge mismatch from the balancing pulse to be safely discharged from the tissue. In order to verify the electrode shorting the peak discharge voltage and exponential time constant of discharge were measured across a 10 nF capacitor which was placed in series with a 2 kΩ resistor as per the protocol used in Sit and Sarpeshkar [38], the results from this test are presented in Fig 6.

The best-fit time of discharge was measured to be <160 μs in both cases. If the shorting phase was deactivated the charge error was observed to push the voltage of the capacitor to the positive rail (+10 V) of the output. This confirms that the charge balancing is operating as designed and the additional passive shorting phase is crucial to maintaining safe charge balancing.

### Saline test

**Fig 7:**
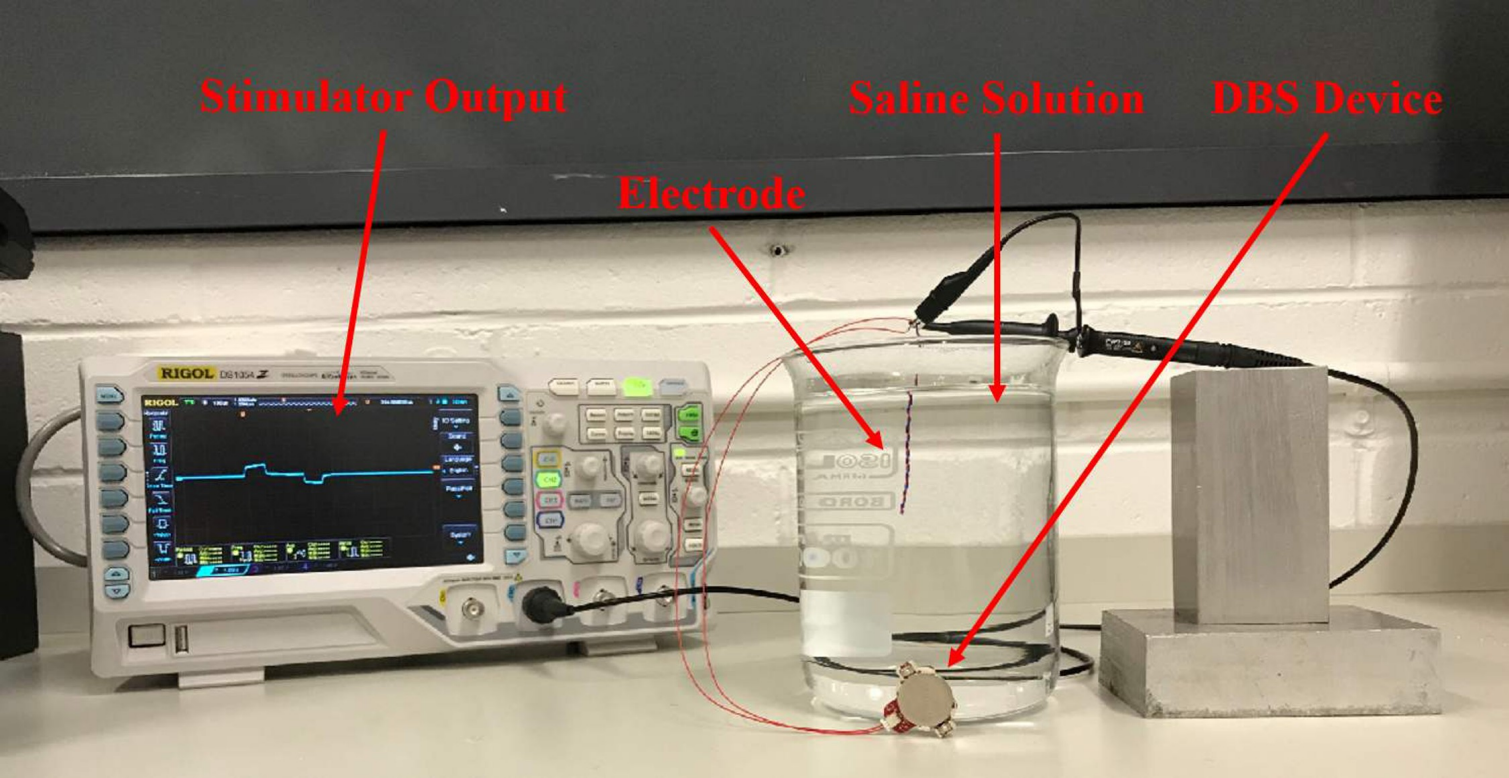
The experimental setup for the saline in-vitro test.

The third test was a stimulation test which was undertaken in the physiological resembling saline, in order to closely replicate the conditions of the neural tissue. The test was conducted by placing a stainless steel 32 AWG twisted wire bipolar stimulating electrode into 1 L of physiologic saline solution (0.9% NaCl). The device was powered by a 220 mAh coin cell battery with stimulation parameters: 130 Hz, 90 μs, 200 μA stimulation and 90 μs, -200 μA charge balancing. A RIGOL DS1054Z 50 MHz Digital Oscilloscope was used to confirm the continuous delivery of the stimulation pulses to the solution, the experimental setup for this test can be seen in Fig 7.

## Discussion

We presented the design and development of a new low-power, programmable, and miniaturized brain stimulation device, with a novel method of active charge balancing. This device has been specifically designed to assist in enabling current and future research in emerging DBS paradigms for pre-clinical animal trials. As such, we aimed to exceed the functionality of comparable preclinical devices, and provide functionality not found in existing preclinical devices.

The device can be produced for less than $10 USD material cost per unit if mass produced, and has been designed in a back-mountable configuration, utilizing a low-cost replaceable battery for operation. Replacing this battery with a rechargeable alternative option would be simple and only require a small redesign to the board to integrate a different battery holder. However, there would be two key disadvantages to this approach: rechargeable cells store less charge than non-rechargeable alternatives, and lithium-polymer batteries are highly volatile if damaged. Particularly in an environment where the animal is not under constant observation, there is a risk that the animal could access and damage the battery, resulting in a fire. This risk is minimised through using a non-rechargeable battery cell with a metallic enclosure.

The key novelty of this work is the fully programmable, low-power and low-cost method of charge balancing. Charge balancing is a key component in any DBS system as it eliminates charge accumulation in the neural tissue and the associated risk of neural damage. This device can output customised waveforms with any amplitude within the output range, with the ability to alter this on the fly. The results in Fig 4 clearly show the ability for this device to produce highly customised biphasic stimulation waveforms, including the Gaussian waveform which some studies have found offers advantages over the traditional rectangular waveform [28]. Additionally, this experiment showed the suitability for this device in delayed feedback stimulation schemes, which many existing devices cannot accomplish.

The evaluation of the device in in-vitro scenarios was presented, clearly demonstrating that the device can accurately and repeatedly output a variety of stimulation waveforms while ensuring the charge injection is balanced. The regulation test clearly showed that the device output is resistant to changes across a variety of common neural tissue loads (~99% regulation) and will reliably stimulate the tissue, even if the impedance changes. The saline test shows the successful operation of the device under conditions replicating those found within the neural tissue.

The device presented is comparable to the equivalent clinical device and exceeds the functionality of nearly all several devices designed for preclinical research in several key categories (Table 1).

**Table 1:** A comparison of DBS systems found in both the preclinical and clinical context.

	Ewing, Lipski [39]	Ewing, Porr [36]	Kouzani, Abulseoud [40]	Pinnell, Pereira de Vasconcelos [5]	Hentall [41]	Kölbl, Kaoua [26]	Medtronic Active SC [42]	Presented Device
Current Amplitude (μA)	-200–200	13–1000	0–200	20–2,000	0–100	26–2,036	0 - 25500mA	-375–250
Minimum Pulse-width (μs)	< 90	<50	< 90	10	100	<60	60	20
Compliance Voltage (V)	3.6 (Battery)	20	3.2 (Battery)	12	34	17	10.5	10
Maximum Frequency (Hz)	185	185	130	5,000	24	300	250	5,000
Lifetime (Continuous Stimulation)	33 Days	10 Days	10 Days	30 Hr	7 Days	6 Days	4 Years+	9 Hr
Charge Balancing	Active	Passive	Passive	Active	Passive	Active	Active	Active
Active Charge Balancing Method	Switched	-	-	Switched	-	Switched	?	Continuous
Non-Standard Waveforms (Gaussian, Sine, Triangular)	No	No	No	No	No	?	No	Yes
Suited for delayed feedback	No	No	No	No	No	?	No	Yes
Channels	2	2	1	2	1	1	1	1
Weight (g)	13.7	11.5	3.2	2.8	1.2	13.8	44	5.0
Size (mm)	24×17×1	33×20×8	12×8	12.5 x 5	15 x 8 x 4	30 x 24 x 14	65×49×15	32.5 × 28 × 8
Mounting	Head or Back	Implant or Back	Head or Back	Head	Implant	Head	Human suitable implant	Back
Programming	Potentiometer	Wired	Wired	Potentiometer	Wired	Wired	Wireless	Wired

A question mark is placed where information is not stated or is unavailable.

## Conclusion

There is clearly still a need for engineering research in the neurostimulation sphere, designing devices, platforms and tools to enable effective research utilising DBS technology. With researchers now investigating new DBS paradigms, there must be devices available to support such research. To address this need, the new DBS device presented herein includes the capability for researchers to more precisely control their output in ways that existing preclinical devices cannot. There is clearly scopes for further work in this area, taking advantage of the advances presented in this work. In particular: in-vivo validation and investigating methods to extend the battery life of the device would have a high priority. Additionally, a clear next step is to integrate this device with a neural recording component to allow for direct evaluation of neurological changes, without relying on behavioural observation. However, these advances must remain closely aligned with the needs of researchers who will be utilizing these devices within their pre-clinical studies.
